# Proposing the VetCompass clinical grading tool for heat-related illness in dogs

**DOI:** 10.1038/s41598-021-86235-w

**Published:** 2021-03-25

**Authors:** Emily J. Hall, Anne J. Carter, Jude Bradbury, Dominic Barfield, Dan G. O’Neill

**Affiliations:** 1 School of Animal, Rural and Environmental Sciences, Nottingham Trent University, Brackenhurst, Southwell, NG25 0QF Notts UK; 2 Queen Mother Hospital for Animals, The Royal Veterinary College, Hawkshead Lane, North Mymms, Hatfield, AL9 7TA Herts UK; 3 Pathobiology and Population Sciences, The Royal Veterinary College, Hawkshead Lane, North Mymms, Hatfield, AL9 7TA Herts UK

**Keywords:** Diagnosis, Prognosis

## Abstract

Heat-related illness is a potentially fatal condition in dogs. Rapid and accurate recognition of the severity can improve clinical management in affected dogs and lead to better outcomes. This study explored retrospective VetCompass veterinary clinical records to investigate the clinical signs recorded for dogs presenting with heat-related illness to primary-care veterinary practice from 2016 to 2018. The relative risk of death associated with these clinical signs was reported and used to develop a novel clinical grading tool. From the clinical records of 856 heat-related illness events, the most frequently recorded clinical signs were respiratory changes (68.73%) and lethargy (47.79%). The clinical signs with the highest relative risk of death were neurological dysfunction, gastrointestinal haemorrhage and bleeding disorders. The novel VetCompass Clinical Grading Tool for Heat-Related Illness in dogs defines three grades: mild (altered respiration, lethargy), moderate (gastrointestinal signs, a single seizure, episodic collapse) and severe (neurological dysfunction, gastrointestinal haemorrhage, bleeding disorders). This novel grading tool offers a simple, evidence-based device to improve recognition of heat-related illness in dogs and promote improved decision-making for earlier interventions such as cooling and hospitalisation. This could improve outcomes and protect the welfare of dogs in the face of rising global temperatures.

## Introduction

Heat-related illness (HRI) is a potentially fatal disorder that affects dogs when their thermoregulatory capacity is overwhelmed, resulting in hyperthermia and subsequent thermal tissue damage^1,2^. There are three recognised main triggers (though with some overlap) of HRI in dogs; exertional HRI occurs following exercise in a hot environment or following intense activity^2,3^, environmental HRI results from exposure to extreme environmental heat or prolonged exposure to a hot environment^1,4^, and vehicular HRI results from either entrapment or travel in a hot vehicle^3^. Both dogs and humans show differing risks for each HRI trigger according to their age, sex, and underlying health status. Young, active male humans^5^ and dogs^3^ are at particular risk of exertional HRI, with breeds including the Labrador Retriever, Golden Retriever, Springer Spaniel and Staffordshire Bull Terrier at greater risk than crossbred dogs^3^. Older dogs and humans have increased risk of environmental HRI^3,6^, in part because elderly individuals are more likely to have underlying health conditions such as respiratory disease and heart failure^7^. Additionally, age-related physiological changes reduce peripheral blood flow and sweat gland function, and thereby limit the effectiveness of homeostatic thermoregulatory functions for effective cooling in older individuals^8^. Vehicular HRI particularly affects young children and babies, most frequently following accidental entrapment^9^. Vehicular HRI can also affect dogs, but brachycephalic (flat-faced) breeds such as the British Bulldog, Pug and French Bulldog appear particularly susceptible^3^, likely because of their inherently reduced ability to thermoregulate resulting from their shortened muzzle and narrowed airways^10^.

The diagnosis of HRI in human medicine traditionally relies upon measurement of body temperature and assessment of neurological function using the “Classical” definitions established by Bouchama and Knochel^1^. These Classical Heat Stroke Criteria define three levels of HRI, rising from mild heat stress (perceived discomfort and physiological strain resulting from mild hyperthermia), to heat exhaustion (moderate illness including weakness, anxiety, fainting, headache and a core temperature that may be elevated up to, but not exceeding 40 °C) and finally heat stroke (severe illness, body temperature exceeding 40 °C accompanied by profound neurological dysfunction including delirium, seizures and coma)^1^. These classical criteria, using a body temperature threshold of ≥ 41 °C, are also used in veterinary medicine for diagnosing and categorising HRI in animals, most frequently dogs^2,11,12^, despite their reliance upon clinical signs/symptoms that, in humans, require verbal communication from the patient e.g. headache, dizziness, anxiety and delirium. However, a study of experimentally induced heat stroke in dogs reported 43 °C as the critical body temperature threshold for inducing clinical, haematological, biochemical and pathological indicators of heat-related illness in dogs^13^. The duration that the dog’s body temperature exceeded 43 °C accurately predicted the risk of death^13^. The lack of consensus in the veterinary literature regarding the critical temperature threshold for HRI in dogs highlights uncertainty in the value and reliability of using body temperature as a diagnostic criterion for HRI.

Following recognition of the limitations from using core body temperature measurement as a diagnostic criterion for the Classical Heat Stroke Criteria in human medicine, a novel HRI staging system was proposed but has not yet been widely adopted for use in humans^14^. Body temperature can fluctuate rapidly in these emergency-care patients and because cooling is recommended as soon as HRI is considered likely, many humans present for medical assessment after they have been cooled and therefore their temperature has already begun to drop. The Japanese Association for Acute Medicine Heat-Related Illness Classification (JAAMHC) has been proposed as an alternative diagnostic and triage tool for use in human patients with HRI^15^. This novel classification included clinical symptoms that were more objectively defined, removed the reliance upon body temperature and self-reported symptoms as diagnostic criteria, and acknowledged that HRI is a progressive rather than a static disorder within patients where increasingly severe pathology is developed with ongoing exposure to heat or failure to receive appropriate treatment (available http://www.mdpi.com/1660-4601/15/9/1962/s1)^15^. Crucially, the JAAMHC novel classification appears better at predicting clinical outcomes for human patients with HRI^15,16^, enabling more appropriate and targeted treatment following initial triage.

Most canine HRI research has applied a variation of the Classical Heat Stroke Criteria when selecting cases for analysis, and generally included only dogs presented to referral hospitals rather than primary-care practices^11,17,18^. This has tended to exclude dogs presenting with milder clinical signs of HRI from inclusion in studies while favouring inclusion of severe cases requiring advanced levels of care, resulting in research findings that may suffer from referral bias^19^. The HRI case fatality rate reported by these earlier referral-based studies ranged from 36 to 50%^11,17,18^, whilst a veterinary primary-care study including dogs with all stages of HRI reported a much lower case fatality rate of 14%^20^, highlighting the benefits from primary-care focused research for results that are more generalisable to the wider dog population. No specific triggers for HRI were reported in the clinical records for almost a third of the dogs presented in the primary-care study^3^, potentially demonstrating a lack of recognition of early signs of HRI by owners or veterinary professionals. Improved awareness of the triggers of HRI, the clinical signs of mild HRI and the actions needed when a dog presents with HRI should be considered as urgent educational priorities for both owners and veterinary professionals to protect canine welfare in the face of rising global temperatures and increasingly frequent extreme heat events^21,22^. Use of the Classical Heat Stroke Criteria definitions that rely on body temperature as a diagnostic criterion may promote continued misdiagnosis because HRI cases that have already begun to cool may be misclassified. This can result in missed opportunities to manage mild or early HRI cases, as noted in human medicine^15^. Therefore, the current paper proposed that a more objective and staged approach for diagnostic criteria should be explored and then used to develop a more reliable HRI clinical grading scheme in dogs.

This study aimed to (i) report the clinical presentations and outcomes of dogs presenting to primary-care veterinary clinics in the UK with HRI, and (ii) adapt the JAAMHC novel HRI classification system to develop a new clinical grading system that is based on canine first opinion data and therefore more reliable and applicable for use in the wider population of dogs. The study is presented in three phases to reflect the sequential nature of the work.

## Phase 1: reviewing the clinical presentation data of dogs affected by heat-related illness

### Data collection and management

This study continues the work previously reported in Hall et al.^3,20^ and used the same dataset described in those studies. The VetCompass Programme collects deidentified electronic patient records (EPRs) from affiliated primary-care veterinary practices in the UK, providing a research database for large scale primary-care studies that includes the clinical records of over 9 million dogs^23–26^. All dogs under veterinary care during 2016 were included in the current study population as previously defined in Hall et al.^20^. Candidate cases for HRI were identified by searching EPRs for the following terms: heat stroke ~ 3, heatst*, hyperthermi*, overheat*, over heated ~ 2, heat exhaustion ~ 2, hot car ~ 2, collapse* + heat, cooling, high ambient temp*. Candidate cases were manually reviewed by two authors (authors 1 and 2) to identify all dogs meeting the case definition of HRI and presenting between 1st January 2016 and 31st December 2018 (see Table 1).

**Table 1:** Case inclusion and exclusion criteria used for heat-related illness (HRI) events in dogs presenting to primary-care veterinary practice, defined in Hall et al.^20^.

Inclusion criteria—Evidence for heat-related illness recorded in the patient record	Final stated diagnosis or insurance claim for a heat-related illness (including heat stroke, heat stress, heat exhaustion or other synonym), and/or History of at least one of the clinical signs listed below, clinical records indicated that these were associated with exposure to these triggers: exposure to a hot environment, physical exertion or both
Clinical signs	• Panting excessively (panting continues despite removal from heat/cessation of exercise) • Collapse not subsequently attributed to another cause (e.g. heart failure, Addison’s) • Stiffness, lethargy or reluctance to move • Gastrointestinal disturbance including hypersalivation, vomiting or diarrhoea • Neurological dysfunction including ataxia, stupor, seizures, coma or death • Coagulation disturbances (bleeding disorders) including petechiae or purpura
Exclusion criteria	Subsequent diagnosis of an infectious or inflammatory condition that was not attributed to primary heat exposure such as kennel cough, pyometra or infectious meningitis HRI or synonym listed only as one of a differential list An earlier diagnosis of HRI that was later revised to exclude HRI, for example the dog was diagnosed with epilepsy following further seizure activity

**Figure 1:**
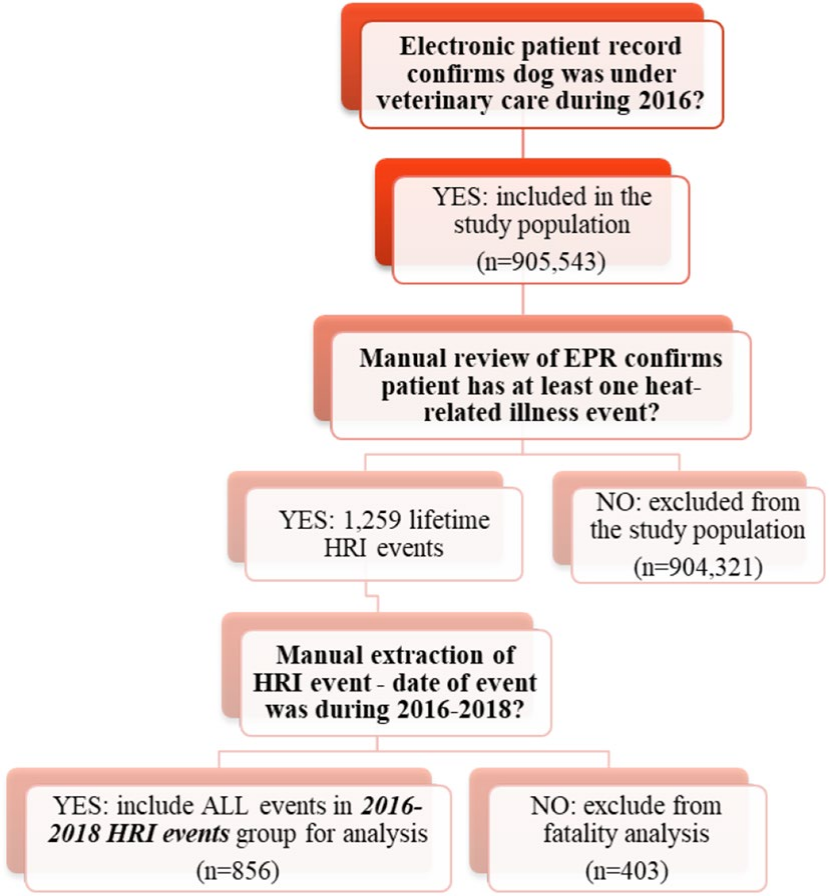
Flow chart of decisions for event inclusion in heat-related illness (HRI) staging analysis and reporting clinical presentations of HRI in UK dogs.

All confirmed HRI events underwent additional data extraction including: date of the event, date of presentation, presenting clinical signs, duration of treatment, and outcome (survived or died including euthanasia, unknown cause of death and unassisted deaths). For this study, only HRI events occurring between 1st January 2016 and 31st December 2018 were included in the analysis, as events that occurred prior to 1st January 2016 resulted in a survival outcome by definition (Fig. 1).

### Investigating the relative risk of death associated with clinical signs recorded for HRI events

The study included 856 HRI events that occurred between 2016 and 2018 identified from the EPRs of 828 dogs (28 dogs had two HRI events in this period), from a population of 905,543 dogs presenting to primary-care veterinary clinics in the UK during 2016. The recorded clinical signs extracted for each HRI event were analysed to determine the relative risk of death overall and specifically for unassisted death (see definitions below) for dogs showing each individual clinical sign compared to dogs not showing that clinical sign:

- Death by any mechanism (including unassisted death, unknown method of death and euthanasia).
- Unassisted deaths only (deaths via euthanasia or an unrecorded mechanism were excluded).

Events without at least one clinical sign recorded in the EPR were excluded from the relative risk analysis. Univariable relative risk analysis using the chi squared test was performed using StatCalc v7.2 (Epi Info, Centre for Disease Control and Prevention, https://www.cdc.gov/epiinfo/index.html). Statistical significance was set at *p* < 0.05.

From the 856 HRI events reviewed, 63 events (7.34%) did not have at least one clinical sign recorded and were removed from the analysis. From the 793 EPRs with at least one recorded clinical sign, the most commonly recorded clinical signs were altered respiration (including excessive panting and dyspnoea) (n = 545, 68.73%), lethargy (n = 379, 47.79%) and intermittent collapse (n = 250, 31.5%). Multiple clinical signs were recorded for 560 HRI events (70.6%).

The clinical signs with the highest relative risk of death overall (including euthanasia, unknown method and unassisted deaths) are shown in Table 2. Dogs presenting with abnormal mentation (including unresponsive, coma, stupor, multiple seizures and status epilepticus), gastrointestinal haemorrhage, petechiae/purpura or ataxia had at least three times the relative risk of death compared to dogs presenting without those clinical signs.

**Table 2:** Proportional fatality and relative risk for **death overall** (including euthanasia, unknown method and unassisted) in dogs recorded with specific clinical signs of heat-related illness cases presenting to primary-care veterinary practices in the UK between 2016 and 2018.

Presenting clinical sign	Clinical sign recorded			Clinical sign not recorded			Relative Risk	95% CI*	*p*-value
	n	Deaths	% Fatality Rate	n	Deaths	% Fatality Rate			
Unresponsive	51	43	84.31	742	47	6.33	13.31	9.85 to 17.98	< 0.001
Coma	15	14	93.33	778	76	9.77	9.55	7.42 to 12.30	< 0.001
Stupor	24	17	70.83	769	73	9.49	7.46	5.33 to 10.45	< 0.001
Multiple seizures	12	8	66.67	781	82	10.5	6.35	4.05 to 9.95	< 0.001
Gastrointestinal haemorrhage	27	14	51.85	766	76	9.92	5.22	3.43 to 7.97	< 0.001
Petechiae/purpura	18	8	44.44	775	82	10.58	4.2	2.41 to 7.32	< 0.001
Ataxia	14	5	35.71	779	85	10.91	3.27	1.58 to 6.80	0.002
Respiratory (excessive panting/dyspnoea)	545	66	12.11	248	24	9.68	1.25	0.80 to 1.95	0.321
Single seizure	30	4	13.33	763	86	11.27	1.18	0.47 to 3.01	0.724
Hypersalivation	33	4	12.12	760	86	11.32	1.07	0.42 to 2.74	0.886
Diarrhoea	76	9	11.84	717	81	11.30	1.05	0.55 to 2.00	0.887
Vomiting	193	22	11.40	600	68	11.33	1.01	0.64 to 1.58	0.980
Collapse-intermittent	250	27	10.80	543	63	11.60	0.93	0.61 to 1.42	0.741
Lethargy	379	21	5.54	414	69	16.67	0.33	0.21 to 0.53	< 0.001

Relative risk describes the risk ratio of death in heat-related illness cases recorded with this clinical sign compared to cases without this clinical sign. *CI confidence interval, n = 793.

The clinical signs with the highest relative risk of unassisted death are shown in Table 3. Dogs presenting with abnormal mentation (including unresponsive, coma, profound depression or multiple seizures) also had significantly increased risk of an unassisted death (*p* < 0.001). Dogs with lethargy recorded as a clinical sign for their HRI event had a significantly reduced relative risk of death (overall and unassisted) (*p* < 0.001).

**Table 3:** Proportional fatality and relative risk for **unassisted death** (excluding euthanasia) in dogs recorded with specific clinical signs of heat-related illness cases presenting to primary-care veterinary practices in the UK between 2016 and 2018.

Presenting clinical sign	Clinical sign present			Clinical sign not present			Relative Risk	95% CI*	*p* -value
	n	Unassisted deaths	% Fatality Rate	n	Unassisted deaths	% Fatality Rate			
Unresponsive	29	21	72.41	709	14	1.97	36.67	20.84 to 64.54	< 0.001
Coma	9	8	88.89	729	27	3.70	24.00	15.51 to 37.13	< 0.001
Stupor	12	5	41.67	726	30	4.13	10.08	4.74 to 21.47	< 0.001
Multiple seizures	7	3	42.86	731	32	4.38	9.79	3.90 to 24.57	< 0.001
Gastro- intestinal haemorrhage	15	2	13.33	723	33	4.56	2.92	0.77 to 11.07	0.115
Single seizure	29	3	10.34	709	32	4.51	2.29	0.75 to 7.05	0.148
Respiratory (excessive panting/ dyspnoea)	508	29	5.71	230	6	2.61	2.19	0.92 to 5.20	0.076
Ataxia	10	1	10.00	728	34	4.67	2.14	0.32 to 14.15	0.429
Petechiae/ Purpura	11	1	9.09	727	34	4.68	1.95	0.29 to 12.98	0.491
Hyper- salivation	31	2	6.45	707	33	4.67	1.38	0.35 to 5.50	0.646
Collapse—intermittent	231	8	3.46	507	27	5.33	0.65	0.30 to 1.41	0.276
Diarrhoea	69	2	2.90	669	33	4.93	0.59	0.14 to 2.40	0.459
Vomiting	176	5	2.84	562	30	5.34	0.53	0.21 to 1.35	0.185
Lethargy	363	5	1.38	375	30	8.00	0.17	0.07 to 0.44	< 0.001

Relative risk describes the risk ratio of death in heat-related illness cases recorded with this clinical sign compared to cases without this clinical sign. *CI confidence interval, n = 738.

### Investigating the relative risk of death associated with presenting body temperature measurements for HRI events

Because body temperature at first veterinary presentation is still routinely used as a key criterion for clinical management of HRI in many veterinary settings^2,11,12,22^, the presenting body temperature of survivors versus non survivors was compared using the Mann-Whitney U test following normality testing showing a non-normal distribution of body temperature values. The relative risk of death associated with several body temperature thresholds at presentation was explored as part of the development process for the current novel VetCompass Clinical Grading Tool for Canine Heat-Related Illness (referred to as the VetCompass HRI Grading Tool hereafter). Three temperature thresholds were compared: < 37.2 °C versus 37.2–40.9 °C, ≥ 41 °C versus 37.2–40.9 °C, and ≥ 43 °C versus 37.2–42.9 °C. For each HRI event, the presenting body temperature was categorised as below, or at/above each of the three thresholds. The relative risk of death overall and the relative risk of unassisted death were calculated for each temperature threshold; HRI events where the presenting body temperature was not recorded in the EPR were excluded from analysis. Dogs that died as a result of euthanasia or unreported mechanisms were excluded from the analysis for relative risk of unassisted death. Associations with hypothermia were also explored because hypothermia has been identified previously as a risk factor for death^11^. The threshold for hypothermia was < 37.2 °C, as defined by Konietschke et al.^27^. Whilst this threshold is lower than the limit (< 37.6 °C) used by Bruchim et al.^11^, this temperature range was derived from a canine population living in a similar climate to the UK.

Body temperature at presentation was recorded for 629 events (73.5%). The median temperature recorded for non-survivors (n = 71), was 41.0 °C (IQR: 39.6–42.4 °C, range: 35.6–43.3 °C) (*p* < 0.001) and was significantly higher than for survivors (n = 558) which was 39.6 °C (interquartile range [IQR]: 38.7–40.5 °C, range: 34.9–43.4 °C). The number of dogs presenting with a body temperature < 37.2 °C, ≥ 41 °C or ≥ 43 °C is shown in Table 4. Dogs presenting with a body temperature ≥ 43 °C had a higher relative risk of death and unassisted death compared to dogs with a temperature ≥ 41 °C (see Table 4). Dogs presenting with hypothermia (< 37.2 °C) did not have a significantly increased relative risk of death (*p* = 0.256) or unassisted death compared to dogs without hypothermia (*p* = 0.256).

**Table 4:** Body temperature at veterinary presentation as a risk factor for death (all causes) and unassisted death in dogs diagnosed with heat-related illness presenting to primary-care veterinary practices in the UK between 2016 and 2018.

Temperature	Clinical sign present			Clinical sign not present			Relative Risk	95% CI*	*p*-value
	n	Deaths	% Fatality Rate	n	Deaths	% Fatality Rate			
< 37.2 °C	14	2	14.29	483	32	6.63	2.16	0.54 to 28.79	0.256
≥ 41 °C	132	37	28.03	483	32	6.63	4.23	2.75 to 6.52	< 0.001
≥ 43 °C	19	14	73.68	596	55	9.23	7.98	5.52 to 11.54	< 0.001

Temperature	n	Unassisted deaths	% Fatality Rate	n	Unassisted deaths	% Fatality Rate	Relative Risk	95% CI	p-value
< 37.2 °C	13	1	7.70	460	9	1.96	3.93	0.54 to 28.79	0.256
≥ 41 °C	110	15	13.64	460	9	1.96	7.06	3.17 to 15.71	< 0.001
≥ 43 °C	12	7	58.33	558	17	3.05	19.15	9.81 to 37.39	< 0.001

Relative risk describes the risk of death in heat-related illness cases meeting each temperature criterion compared to cases that did not meet this criterion. *CI confidence interval n = 629 for overall death analysis, n = 583 for unassisted death analysis.

## Phase 2: adapting the JAAMHC staging criteria for use in canine HRI patients

The clinical symptoms identified in the JAAMHC staging criteria for human HRI were adapted for application to dogs in the current study (see Table 5) to create the novel VetCompass HRI Grading Tool. This adaptation was based on differences in physiology between the two species (namely thermoregulatory reliance on panting rather than sweating in dogs) and the evidence base generated from the relative risk analysis for presenting clinical signs reported above. The clinical signs that were associated with significantly increased risk of death overall and risk of unassisted death in the current study (*p* < 0.05) were included in the most severe category, notably: altered mentation (including unresponsive, coma, profound depression, multiple seizures and ataxia) gastrointestinal haemorrhage and petechiae/purpura.

**Table 5:** Development of the novel VetCompass Clinical Grading Tool for Heat-Related Illness in dogs, based upon the JAAMHC human heat-related illness scale.

Japanese Association of Acute Medicine Heat-Related Illness Classification stages	Human symptoms	VetCompass Clinical Grading Tool for Heat-Related Illness in dogs grades	Adapted canine clinical signs
Stage I	Dizziness, faintness, slight yawning Heavy sweating Muscle pain, stiff muscles (muscle cramps) Impaired consciousness is not observed	Mild	No impaired consciousness observed Lethargy or stiffness Panting heavily or respiratory distress
Stage II	Headache, vomiting, Fatigue, sinking feeling Declined concentration and judgement	Moderate	Vomiting and/or diarrhoea (no blood present), hypersalivation Collapse (intermittent only) A single seizure
Stage III	Includes at least one of the following: Central nervous system manifestation (impaired consciousness, cerebellar symptoms, convulsive seizures) Hepatic/renal dysfunction Coagulation disorder	Severe	Includes at least one of the following: Central nervous system impairment (ataxia, two or more seizures, profound depression, unresponsive, coma) Hepatic/renal dysfunction (confirmed on blood tests) Gastrointestinal haemorrhage Coagulation disorder
Unreported	N/A	Unreported	No clinical signs or diagnostic results recorded in the clinical history

Although the current study did not evaluate laboratory diagnostic results, a previous study of post mortem findings in dogs that died as a result of HRI reported hepatomegaly in all cases, and hepatic parenchymal necrosis in over half the dogs examined^28^. Renal pathology was also noted in all the dogs examined, ranging from interstitial and glomerular congestion to tubular necrosis in both the proximal and distal convoluted tubules^28^. Abnormal renal and hepatic function were therefore retained in the diagnostic criteria for severe HRI in dogs.

## Phase 3: retrospective grading of 2016–2018 canine HRI events using the novel VetCompass grading tool

### Analysis

All HRI events identified during 2016–2018 were retrospectively classified according to the novel VetCompass HRI Grading Tool defined in Table 5. Where an HRI event had insufficient information recorded in the EPR to allow classification, the event was categorised as “unreported” as per the definition in Table 5. Descriptive statistics and the distribution of cases were compared with the distribution of human HRI events.

The utility of temperature thresholds was explored within the VetCompass HRI Grading Tool. The inclusion of temperature threshold ≥ 43 °C as a severe grade criterion within the VetCompass HRI Grading Tool would have changed the grading of three events: one originally graded mild would have changed to severe and two events originally graded moderate would have changed to severe. None of these events resulted in fatality, and only one of the three events resulted in hospitalisation beyond one day. This suggests limited additional value from adding a temperature threshold ≥ 43 °C as a severe grade criterion to the grading tool.

The inclusion of temperature threshold ≥ 41 °C as a severe grade criterion within the VetCompass HRI Grading Tool would have changed the grading of 83 (9.70%) of the 856 events; 40 (12.62%) of the 317 events originally graded mild would have changed to severe, and 43 (11.75%) of the 366 events originally graded moderate would have changed to severe. From the 40 events originally graded mild, there was one fatality, an elderly animal the owners elected to euthanise. From the 43 dogs originally graded moderate, there were four fatalities—three of which were unassisted deaths. Of these four dogs, one elderly dog was euthanised due to underlying laryngeal paralysis, one of the unassisted deaths was a dog that had experienced multiple HRI events that year and died under sedation, the remaining two dogs were brachycephalic one of which experienced a cardiac arrest and the other died following a tracheostomy. There were an additional 17 events that resulted in hospitalisation beyond one day, however all these events were discharged after one overnight stay.

This review suggests that the application of temperature thresholds add little additional value to the grading tool and therefore body temperature was omitted from the final VetCompass HRI Grading Tool definitions, in line with similar recommendations from Yamamoto et al.^14^ for human HRI patients.

### The distribution of HRI event severity using the novel VetCompass grading tool

**Figure 2:**
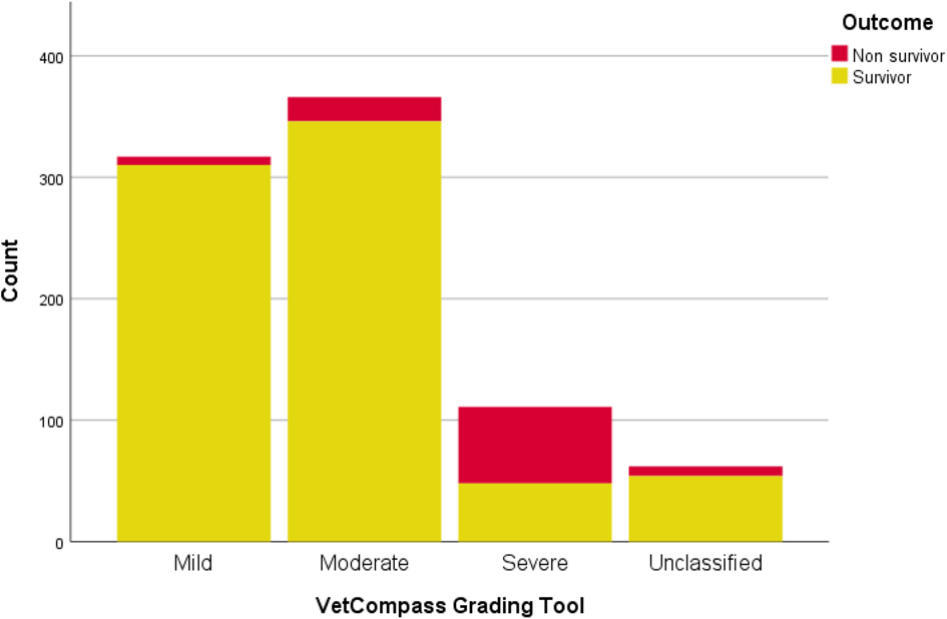
The event distribution and survival outcomes for HRI grades defined using the VetCompass Clinical Grading Tool for Heat-Related Illness in dogs presenting to primary-care veterinary practices in the UK between 2016 and 2018.

The distribution of HRI events using the VetCompass HRI Grading Tool is shown in Fig. 2. Overall, 39.92% of classified HRI events were graded mild, 46.10% graded moderate and 13.98% graded severe. In comparison, the distribution of human HRI events presenting to 102 Japanese hospitals during the summer of 2012 classified by the JAAMHC in one paper was 48.86% stage I, 33.24% stage II and 17.90% stage III^15^. The distribution of canine HRI events between the grades of the VetCompass HRI Grading Tool closely reflected the distribution in human HRI patients reported by Yamamoto et al.^15^.

### Comparing the fatality rate distribution of canine versus human HRI events

The fatality rate (death overall) for canine HRI events classified using the novel VetCompass HRI Grading Tool was 2.21% for mild events, 5.46% for moderate events and 56.76% for severe events. The unassisted death fatality rate increased from 0.32% in mild events, to 2.19% in moderate events, and 23.42% in severe events.

These fatality rates compare to the findings of a human study using the JAAMHC classification for human HRI events: stage I patients all survived, the fatality rate for stage II patients was 0.7%, increasing to 10.2% for stage III patients^15^.

### The final VetCompass HRI grading tool

**Figure 3:**
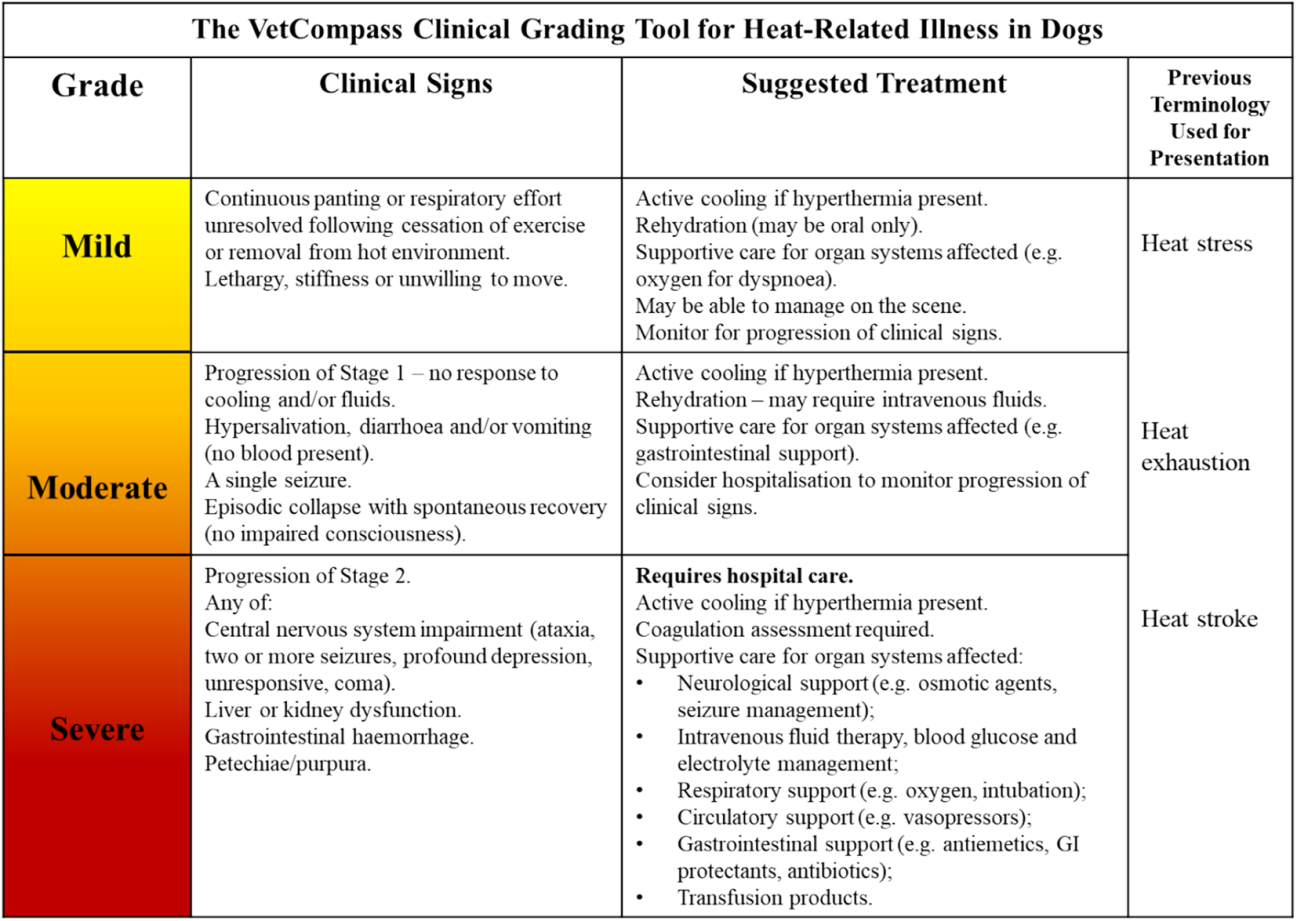
The novel VetCompass clinical grading tool for heat-related illness in dogs.

The final VetCompass Clinical Grading Tool for HRI in dogs (Fig. 3) incorporated clinical signs but excluded presenting body temperature as a defining criteria. Although a presenting body temperature ≥ 41 °C and ≥ 43 °C were both associated with a significantly increased relative risk of death compared to dogs presenting with lower body temperature, it is the duration of temperature elevation that results in clinical pathology, so a single temperature reading alone is poorly diagnostic of severe illness unless that temperature is above 45 °C^13^. Clinical management should aim to prevent worsening of the patient’s condition through early temperature management, fluid therapy and supportive management of body systems affected^29,30^.

### Ethics approval

Ethics approval was granted by the RVC Ethics and Welfare Committee (reference number SR2018-1652). This project used only data from a research repository, no actual dogs were involved in the project.

## Discussion

This study reports that the most commonly recorded clinical signs in UK dogs with HRI presenting to primary-care veterinary practices were respiratory changes (excessive panting/dyspnoea) and lethargy. This is a key finding to assist dog owners, as early recognition of these milder clinical signs could allow them to take earlier action to prevent worsening of their pet’s condition and prevent progression of the HRI. We also report that the clinical signs associated with the highest relative risk of death (both overall and unassisted) were abnormal mentation including unresponsive, coma, stupor and multiple seizures (including status epilepticus). The results from relative risk analysis were applied as an evidence base to support an adaptation of the JAAMHC classification for human HRI events to create the novel VetCompass Clinical Grading Tool for HRI in dogs.

Previous veterinary studies of HRI in dogs have tended to focus on the most severe subset of cases, using the Classical Heat Stroke Criteria heat stroke definition to determine case inclusion criteria^11,18,31^. These studies were generally restricted to dogs referred for specialist care, and so are poorly representative of the overall canine population. For this reason, we elected to compare the distribution of canine HRI events in the current study to the distribution of human HRI events reported in a study from Japan^15^ rather than to previously reported studies in dogs. The progressive nature of HRI should theoretically result in the majority of HRI events being classed as stage I, with fewer events classed as stage II, and the minority of events progressing to stage III, especially as public awareness of HRI has increased the likelihood of early interventions such as cooling and removal from hot environments^32^. More canine HRI events were classified as moderate than mild compared to the human results, which may represent a variety of owner-related factors. Increased public awareness of HRI through national campaigns may have resulted in more owners managing milder cases at home rather than seeking active veterinary care. The impacts from financial costs of veterinary consultation may also play a role here: owners may preferentially present more severe cases for veterinary care because they perceive the clinical risk justifies the financial outlay whereas they may choose to monitor and manage milder cases on their own. This could drive a relative underreporting of milder HRI in UK dogs. It is also possible that some owners either did not recognise mild HRI events, or as suggested by Packer et al.^33^, brachycephalic owners may in fact perceive mild HRI as “normal for their breed of dog”.

The clinical signs included in the Grading Tool for severe HRI reflect the pathological findings reported in a study of 11 dogs that died (ten unassisted deaths and one euthanasia) as a result of HRI in one referral hospital^28^, namely evidence of a coagulopathy, gastro-intestinal mucosal necrosis, renal damage, hepatopathy and brain damage. The presence of petechiae/purpura suggests a coagulopathy had developed, a consequence of endothelial damage from both thermal injury to cells and also secondary hypoperfusion, shock and systemic inflammatory response^1,28^. Disseminated intravascular coagulopathy (DIC) is a life threatening syndrome that occurs when widespread activation of the coagulation system is triggered alongside dysregulation of thrombin generation (e.g. a hypercoagulable to a hypocoagulable state)^34^. Heat-related illness can trigger DIC, as reported by Bruchim et al. where all of the dogs examined post mortem were found to have developed DIC as a result of their HRI^28^. A mortality rate of 62.5% has been reported for dogs with overt DIC (three or more coagulation variable abnormalities detected during clinical screening) triggered by a variety of inciting disorders including HRI^35^. It is therefore appropriate that the clinical signs of petechiae/purpura were included in the criteria for severe HRI in dogs, as it is for humans^14^.

Histopathological examination of the brains and meninges of dogs that died as a result of HRI revealed mild to severe oedema and hyperaemia in all sections examined from all dogs in the previous study^28^. In another study of dogs presenting to a referral hospital with HRI, cases presenting with profoundly altered mentation (obtunded or comatose) had a fatality rate of 70%, and the dogs that presented with disorientation or stupor had a fatality rate of 41%^11^. In the present study, all clinical signs indicative of altered mentation had a significantly increased relative risk of both death overall and unassisted death, and are therefore included in the severe HRI criteria in the current grading tool.

Altered respiration was the most frequently recorded clinical sign for all dogs with HRI in the present study. Pulmonary oedema and hyperaemia were noted in all the deceased dogs examined at post mortem by Bruchim et al.^28^, and tachypnoea was reported in around 80% of the dogs presented to the same referral hospital with heat stroke^11^. However, altered respiration including panting heavily or respiratory distress, was not associated with an increased relative risk of death or unassisted death in the present study. As panting is a key thermoregulatory mechanism in dogs for cooling, this clinical sign will be present in all conscious dogs with hyperthermia at some point during a HRI event and is therefore included in the criteria for mild HRI in the grading tool. Comatose dogs cease panting, which likely contributes to their reduced rate of cooling when immersed in cold water^36^, and potentially contributes to the increased fatality rate in dogs that developed impaired mentation as a result of HRI.

The findings of the present study support the recommendations of recent human HRI studies^14,15^ that body temperature should no longer be considered a reliable diagnostic criterion for staging HRI in dogs. Body temperature can fluctuate rapidly, especially when active cooling methods have been applied prior to clinical assessment^37^. There are also conflicting results on the associations between body temperatures per se and pathological changes in dogs. In a study of anaesthetised dogs, prolonged (90 min) whole body hyperthermia at 42.5 °C failed to induce histopathological or clinically significant neurological disturbance, when other physiological responses to hyperthermia (for example respiratory alkalosis or reduced blood pressure) were prevented^38^. Post exercise body temperatures of 42.5 °C have also been reported in dogs showing no clinical signs of HRI^39,40^. Experimental heat stroke studies carried out on dogs in the 1970s suggested that 43 °C is the critical temperature threshold for canine HRI^13^. In a series of inhumane experiments that involved exposing dogs to ambient temperatures of 50 °C both with and without physical exertion until they collapsed, a body temperature of 43 °C was reported as the critical limit for clinical effects. Dogs that did not exceed a body temperature of 43 °C had no clinical or clinicopathological signs of HRI, whilst dogs that developed a body temperature > 44 °C all died of heat stroke. The longer a dog had a body temperature > 43 °C, the greater the likelihood of death. The previously proposed critical body temperature threshold of > 41 °C for diagnosis of HRI can therefore no longer be considered appropriate. Any dog with a temperature approaching 41 °C should be actively cooled as a matter of urgency, but clinical signs should be used to then determine the severity of HRI present.

Hypothermia on presentation has previously been reported as being associated with an increased fatality rate^18^. However, Drobatz and Macintire^18^ clarified that it was not possible to determine if hypothermia due to cooling contributed to the poor outcome, or if hypothermia was instead an effect of poor tissue perfusion caused by profound clinical disease. In the present study, hypothermia on presentation was not associated with an increased risk of death overall or unassisted death, reflecting the findings of Bruchim et al.^11^. Many veterinary texts caution against aggressive, rapid cooling of dogs with HRI due to a perceived hazard of hypothermia^41–43^. However, from the current study, it appears hypothermia does not increase the fatality risk for dogs. In humans with exertional HRI, cold water immersion is the gold standard treatment^44^. Rapid (within minutes of collapse) initiation of cooling using cold (10 °C) water immersion until the patient’s body temperature dropped below 38.8 °C was associated with 100% survival rate in one study of over 200 HRI events^37^. In dogs, work to date has included the use of warm water (30 °C) immersion to effectively cool dogs with exertional hyperthermia but not HRI^45^, and dogs with experimentally induced HRI cooled with a range of water temperatures^36^. The latter study reported that tap water (15–16 °C) achieved the fastest rate of cooling in conscious dogs with HRI, but found that comatose dogs cooled at a slower rate due to the cessation of panting^36^. As drowning is a risk to any HRI patient undergoing water immersion, especially patients with central nervous system impairment, constant monitoring is needed to ensure the head remains above the water level^29,44^.

A novel severity scoring system has been proposed previously for dogs with HRI by Segev et al.^46^. However, that scoring system required comprehensive laboratory analysis including prothrombin and activated partial thromboplastin time, blood glucose, biochemistry, and haematology analysis, and required the use of a statistical model to determine the overall patient score. This reliance on laboratory tests requires the dog to be physically presented to the veterinary practice in order to take the samples, and the owners to be both willing and able to pay for the tests before the score can be determined. As noted in that study’s limitations, the scoring system proposed by Segev et al. is aimed at assisting decision making for only the most severe of cases, and requires further testing before it can be deemed a reliable model^46^.

In contrast, the novel VetCompass HRI Grading Tool proposed in the present study aimed to create a grading system that could be used by veterinary professionals to triage patients over the telephone or when presenting to the veterinary practice, and that can be used without the need for complex statistical modelling, potentially expensive laboratory testing or indeed any mathematical calculations. The tool could be displayed as a poster (see Fig. 3) in veterinary receptions, and be added to the emergency or treatment room as a quick reference guide to assist veterinary staff with decision making and provide advice to owners regarding a potential prognosis for dogs with HRI. It could be adapted as an educational tool to improve public awareness of the early signs of HRI in dogs, to be shared on social media to get to the attention of owners who are the key decision-makers in the early phases of most dogs with HRI. Delayed cooling has been associated with an increased fatality rate in dogs presenting to a referral hospital with HRI^11^, thus improving owners’ ability to recognise and promptly respond to signs of HRI in dogs should be considered a priority as global temperatures continue to rise.

This study had some limitations. As noted in previous studies, VetCompass clinical record data are not recorded for research purposes^3,20^. As a result, there are missing data within the dataset and descriptive data may be inaccurate or incomplete, because they are reliant upon the history recorded by the attending veterinary surgeon and so can be impacted by factors such as stress and workload, especially when recording clinical notes from emergency patient presentations. For events with an unassisted death, progressive changes in clinical signs may not have been accurately recorded within the electronic clinical history but may instead have been recorded on alternative documentation such as paper-based hospital records or cardiac arrest monitoring charts. Likewise, HRI cases that were euthanised may have limited clinical histories and clinical signs recorded if the owner requested euthanasia early during presentation. Some cases may also have been euthanised before additional clinical signs developed if there were financial constraints on treatment options, or if the dog was elderly or had other underlying health conditions that contributed to the decision to euthanise.

Future evaluation may help to refine the VetCompass HRI Grading Tool further by exploring the tool’s predictive ability for other HRI factors such prolonged hospitalisation and dog conformations.

## Conclusion

This study presents the novel VetCompass Clinical Grading Tool developed for dogs with heat-related illness, and aims to improve both owner and veterinary recognition of the progressive clinical signs associated with the disorder. Continued use of the previously available Classical Heat Stroke Criteria should be done with caution because those criteria were not designed for use with canine patients. There is a risk of underestimating the severity of the dog’s condition due to a reliance on patient reported symptoms and presenting body temperature rather than observed clinical signs. Continued use of body temperature as a diagnostic criterion cannot be supported, in line with recent human medical advances. The VetCompass HRI Grading Tool offers a quick, practical and evidence-based tool for veterinary professionals to grade HRI cases and should assist in optimising clinical care and the clinical outcomes. As global temperatures continue to rise, uncomplicated triage tools such as the one proposed in this study can help to improve owner understanding and assist in decision making for effective early management when HRI occurs.

## Data Availability

Original data used for the current study is available: https://researchonline.rvc.ac.uk/id/eprint/13572/.

## Abbreviations

CI: Confidence interval
EPR: Electronic patient record
HRI: Heat related illness
